# Minimizing risks and monitoring safety of an antenatal care intervention to mitigate domestic violence among young Indian women: The *Dil Mil* trial

**DOI:** 10.1186/1471-2458-12-943

**Published:** 2012-11-01

**Authors:** Suneeta Krishnan, Kalyani Subbiah, Prabha Chandra, Krishnamachari Srinivasan

**Affiliations:** 1 Women’s Global Health Imperative, RTI International, 114 Sansome Street, Suite 500, San Francisco, CA, 94104, USA; 2 St. John’s Research Institute, St. John’s National Academy of Health Sciences, Koramangala, Bangalore, 560034, India; 3 National Institute of Mental Health and Neurosciences, Hosur Road, Bangalore, 560029, India

**Keywords:** Domestic violence, Women’s empowerment, Randomized controlled trial, India

## Abstract

**Background:**

Domestic violence - physical, psychological, or sexual abuse perpetrated against women by one or more family members – is highly prevalent in India. However, relatively little research has been conducted on interventions with the potential to mitigate domestic violence and its adverse health consequences, and few resources exist to guide safety planning and monitoring in the context of intervention research. *Dil Mil* is a promising women’s empowerment-based intervention developed in India that engages with young women (daughters-in-law) and their mothers-in-law to mitigate domestic violence and related adverse health outcomes. This paper describes the design of a randomized controlled trial of *Dil Mil* in Bengaluru, India, with a focus on strategies used to minimize study-related risks and monitor safety.

**Methods/design:**

A phase 2 randomized controlled trial using a parallel comparison of the *Dil Mil* intervention versus standard care will be implemented in three public primary health centers in Bengaluru. Young pregnant women in the first or second trimester of pregnancy will be recruited from antenatal services at study health centers and through community outreach. If eligible and willing, their mother-in-law will also be recruited. Once enrolled, dyads will participate in a baseline interview and then randomized either to the control arm and receive standard care or to the intervention arm and receive standard care plus the *Dil Mil* intervention. Additional evaluations will be conducted at 3 months and 6 months postpartum. Data will be analyzed to examine the feasibility and safety of the intervention and the effect of the intervention on intermediary outcomes (the empowerment of daughters-in-law and mothers-in-law), incidence of domestic violence among daughters-in-law, and health outcomes including perceived quality of life, psychosocial status and maternal and infant health outcomes.

**Discussion:**

This study offers approaches that may help guide safety planning and monitoring in other domestic violence intervention trials in similar settings. Moreover, given the staggeringly high prevalence of domestic violence against young women in India (and indeed globally) and the dearth of data on effective interventions, this study is poised to make an important contribution to the evidence-base for domestic violence prevention.

**Trial registration:**

ClinicalTrials.gov Identifier: NCT01337778

## Background

A large number of studies have documented the occurrence of domestic violence (DV) - physical, psychological, or sexual abuse perpetrated against women by one or more family members - globally and in low- and middle income country (LMIC) settings such as India [1-11]. However, relatively little research has been conducted on interventions with the potential to mitigate DV and its adverse health consequences, and few resources exist to guide safety planning and monitoring in the context of intervention research. In this paper we describe the design of a randomized controlled trial of an intervention to mitigate DV and related adverse health consequences in antenatal care settings in urban Bengaluru, India, focusing on the strategies used to address ethical challenges in research implementation.

Randomized controlled trials of social and behavioral interventions are growing in number. However, there is little guidance regarding how best to promote safety of participants enrolled in these studies – including how to define, track and monitor safety risks and adverse events. This is especially the case for research on interventions to prevent and/or mitigate DV [12,13]. Over a decade ago, the World Health Organization (WHO) issued its recommendations on ethics and safety in research on domestic violence against women [14,15]. The WHO guidelines emphasized the importance of promoting respondent and interviewer safety (for example, by ensuring privacy during interviews and interviewing only one woman in each household), careful training of research staff to ask questions and respond to disclosures about DV, offering referrals to critical support services (e.g., including a counselor or consultant on the study team, linking to a local counseling center, providing a directory of referrals services), and ensuring that evidence is used to advance policy and program development.

Several researchers have described their attempts at applying the WHO’s guidelines in LMIC contexts [15-19]. Andersson et al., in their cross-sectional survey of DV in Pakistan, had two interviewers visit each household, with one interviewer engaging with a husband or mother-in-law while a second interviewer spoke with the primary female respondent [16]. Interviewer safety was promoted by forming a relatively large field team, including male staff, and organizing transport and accommodation for the teams during data collection. Special efforts were also made to enhance rapport with respondents. Interviewers were encouraged to recollect abuse that had happened to themselves or someone close to them and to think about how difficult disclosures can be. In the interviews, staff used introductions such as “I know how hard this is to talk about” as a preface to questions regarding respondents’ experience of violence.

The literature on ethical concerns in DV research from LMICs has primarily focused on issues arising in cross-sectional surveys. To our knowledge, this paper is the first to describe the ways in which ethical concerns shaped the design and implementation of a randomized controlled trial of an intervention to reduce DV among young women in a LMIC setting. Given the high prevalence of DV and young married women’s limited decision-making autonomy in India, risk analysis and minimization were a necessary part of the process of designing our study. Risk analysis involved using the published literature and our prior research to assess the nature, likelihood and severity of harms that may arise from participation in the proposed trial and was followed by identification of strategies to minimize risks and assessment of whether potential benefits of participation outweighed potential risks. The goal of this process was to ensure that our research did not inadvertently heighten women’s risk of experiencing DV. Below we describe the trial design and implementation as it was shaped by these ethical concerns.

### The Dil Mil intervention: context and rationale

Marriage is a social necessity in India, and the vast majority of reproductive age women are married by age 25 to 29 years. Marriages continue to be arranged by elders and there are strong social pressures on parents to marry their daughters young. Although the legal minimum age for marriage is 18 years for women, in 2007–9 almost half (43%) of women aged 20–24 years were married before 18 in India [20]. Karnataka is one of the states with the highest incidence of child marriage in the country; 50% of 20–24 year old women were married before 18 [21]. Once married, women face considerable pressures to prove their fertility [22-25]. Moreover, they have limited decision-making autonomy within marriage, and are highly vulnerable to DV. In our longitudinal study with 747 women ages 16 to 25 years living in low-income neighborhoods of Bengaluru, the capital of Karnataka, 56% of participants reported experiencing physical violence perpetrated by their husband at baseline, and over half of these women also reported incident violence during a 2-year follow-up period [26]. Notably, the majority (77%) of the cohort reported ever having experienced physical, psychological and/or sexual DV perpetrated by husbands, mothers-in-law (MILs), or other members of the marital family (unpublished observation, Krishnan). A representative sample survey of reproductive age women in Karnataka has suggested that abuse begins early in marriage: 80% of respondents with a history of DV reported experiencing physical violence within the first five years of marriage [27]. Studies have also documented that DV continues during pregnancy and the perinatal period, and is associated with an array of adverse maternal and child health outcomes [11,28-33].

DV is widely tolerated, and both women and men consider violence to be justified in response to perceived violations of social expectations of a “good” wife or mother [34]. Conflict and violence are a private, family matter, and not surprisingly, we found that few women sought extra-familial sources of support (unpublished observation, Krishnan). Awareness regarding the Protection of Women from Domestic Violence Act, 2005, which provides a comprehensive definition of DV and a range of actions to promote women’s safety and rights, remains low [35]. Moreover, few concerted efforts are being made to increase public knowledge regarding the law, and there is little coordination among service providers.

We conducted qualitative research with participants of our longitudinal study to identify feasible and acceptable approaches to mitigating DV and promoting women’s health [36]. Focus group discussions and interviews revealed that MILs played a key role in instigating DV. However, women also acknowledged that that they were better able to cope with violence when they had a supportive MIL. In fact, women noted that MIL’s support was preferable to natal family support because husbands were less likely to retaliate in response to interventions by their own mother compared with individuals from their wife’s family. Overall, women clearly expressed that MILs can take a leadership role in reducing DV in the family, laying a rationale for the proposed intervention. Based on these and additional insights from the published literature [37-39], we developed the intervention, *Dil Mil* (meaning *“Hearts Together”* in Hindi, India’s national language), which engages daughters-in-law (DIL) and MILs to mitigate DV and related adverse health outcomes.

### The Dil Mil trial: aims

We are testing the *Dil Mil* intervention through a phase 2 randomized controlled trial. The specific aims of the trial are to: 1) assess feasibility and safety of the intervention by monitoring and assessing recruitment, adherence to study visits, retention, contamination across arms, and the incidence of adverse events; 2) evaluate the potential effectiveness of the intervention by examining the effect of *Dil Mil* on empowerment of DILs and MILs (intermediary outcomes) and 3) examine preliminary evidence of the impact of the intervention on DV incidence and related health outcomes (perceived quality of life, psychosocial status, and maternal and infant health) among DILs.

**Figure 1:**
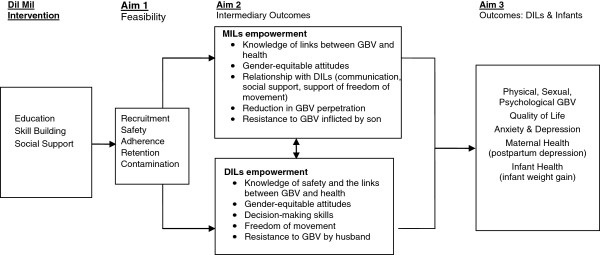
***Dil Mil *Causal Model.**

Figure 1 depicts the causal model. We hypothesize that the *Dil Mil* intervention is feasible and can be delivered safely, leading to increases in DILs’ knowledge about safety and the links between DV and health, gender-equitable attitudes, decision-making skills, and ability to resist DV (DILs’ empowerment) and in MILs’ knowledge, gender-equitable attitudes, and communication and provision of social support to DILs including their resistance to violence (MILs’ empowerment). We posit that these changes will reduce DILs’ risk of DV and related adverse health outcomes. Based on the results of this phase 2 trial, we will refine our approach and determine the merit of a future phase 3 trial of the *Dil Mil* intervention.

## Methods/design

### Study setting and design

Our research is being conducted in Bengaluru, the third most populous city in India with a population of over 8.5 million [40]. Among urban women aged 18 to 29 in the state of Karnataka, about 30% were married by age 18 and 17% had received no schooling [2]. Antenatal care utilization is high; 71% of urban women had their first antenatal care visit in the 1st trimester [2]. The municipal government of Bengaluru operates a network of 36 urban primary health centers, all of which offer antenatal and postpartum services [41]. These centers are located in the middle of clusters of lower income, working class neighborhoods with populations ranging between 60,000 to 90,000. Women residing in these neighborhoods engage in home-based work or are employed as domestic workers or in the construction or garment industry, and men are involved in a range of unskilled and semi-skilled occupations such as manual labor, masonry, and carpentry.

**Figure 2:**
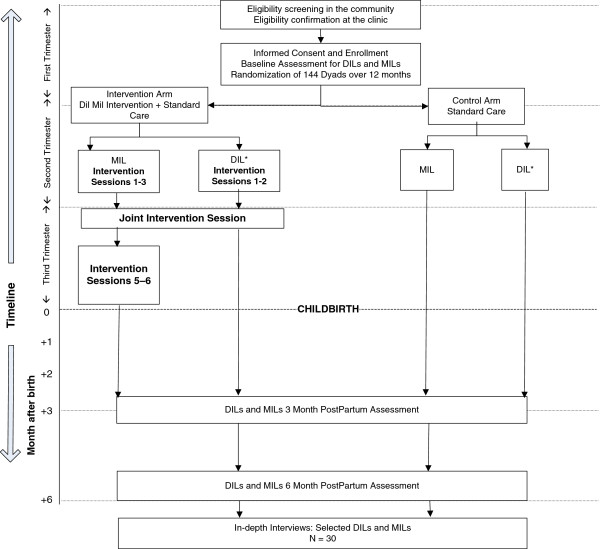
Overview of Study Procedures.

A phase 2 randomized controlled trial using a parallel comparison of the *Dil Mil* intervention versus standard care will be implemented in three public primary health centers in Bengaluru. Figure 2 describes the flow of study activities. Table 1 summaries the ways in which the design responds to ethical challenges involved in research on interventions to mitigate DV. DIL-MIL dyads will be randomized either to the control arm and receive standard care or to the intervention arm and receive standard care plus the *Dil Mil* intervention. Our prior research in these centers showed that risks are minimized when research is conducted in this setting since accessing care at the centers is well accepted in the community and there is little scrutiny of activities conducted within their four walls.

**Table 1:** Overview of ethical challenges and design responses

**Study Component**	**Ethical Challenges**	**Design Response**
Definition of study population	Pregnant women and women with a history of domestic conflict and violence are vulnerable groups	Antenatal care offers a potentially safe opportunity to intervene on violence; Interventions to mitigate family conflict and violence may have beneficial impact on pregnancy outcomes
Study advertisement	Disruption of family relationships because of family members’ perception of the intervention as being focused on DV or because of resistance to the types of social and behavioral change encouraged by the intervention, and risk of backlash from family and community	Study framed as testing a family health intervention to promote the health of younger and older women and children; Community advisory group comprising community elders and leaders (male and female) formed and community meetings held to enlist support for the study and introduce research team
Participant recruitment	Limited decision-making autonomy among young women; Loss of confidentiality due to screening for domestic conflict and violence; Loss of confidentiality during participant follow-up	Multistep recruitment process used beginning with DILs; Completion of eligibility screening in private room at health center; Ascertainment of acceptable modes of contacting participants for follow-up; Use of standardized scripts to respond to questions from family/community members and avoidance of discussions about study specifics with non-participants; Avoidance of conversations that may jeopardize confidentiality in public settings
Participant retention and follow-up	Loss of confidentiality	Use of participant tracking system with modes of contact approved by participant; Staff trained not to discuss sensitive study information with participant or others in public settings; Use of standardized scripts for participant follow-up
Safety Monitoring	Defining safety in a context of high prevalence of DV and in a population with prior history of family conflict and violence; Monitoring safety	Classification of safety-related events as “safety alerts” or “adverse events,” with alerts comprising incidents that are highly unlikely to be related to study participation and/or not considered to be severe and adverse events comprising incidents that are unexpected, temporally associated with and likely to be related to study participation and severe; Establishment of rapport with participants and creating a safe environment at the health center by offering information on DV-related resources at every visit, establishing an information desk at study health centers, and providing staff contact information; Presence of on-site counselor
Randomization	Confusion regarding or lack of understanding of randomization	Explanation of randomization in terms of a lottery; Opening of envelope with treatment assignment in front of dyad
Intervention implementation	Stress, discomfort or distress as a result of intervention participation; Loss of confidentiality in group education sessions	Establishing ground rules for group session participation including respect for each other’s views; Inclusion of activities to promote peer support and dialogue, such as co-counseling; Staff trained to handle difficult situations arising during group sessions; Counseling available on site and referral information offered; Child care and food offered; Participants and staff take a pledge of confidentiality regarding personal information shared in the group
Data collection	Stress, discomfort or distress caused by interview questions; Loss of confidentiality	Interviewers trained to conduct interviews with sensitivity and empathy; Use of statements such as, “some women can feel sad or upset by these questions—do remember that you can decide not to answer any question” repeated at strategic points of interview; Counseling available on site and referral information offered; All interviews conducted in closed room at health center; Data identified only through unique numeric identifiers

The research team will be comprised of female intervention facilitators and research interviewers with a minimum of five years of experience in conducting qualitative and quantitative research on DV and women’s health. In fact, all staff were involved in the formative longitudinal research that led to the development of the *Dil Mil* intervention. Moreover, team members live in or near the study communities or communities with similar socio-cultural characteristics. Care will be taken to ensure that staff do not handle study activities such as interviews or intervention session facilitation if they involve participants who are from their neighborhood or within their social circle to minimize the risk of breaches in confidentiality. All staff will undergo training on research ethics and sign an agreement to keep all study participant-related information confidential.

The study protocol has been reviewed and approved by the institutional review boards of RTI International in the United States and the St. John’s Medical College in Bengaluru, India.

### Study population

As described in Table 2, the *Dil Mil* trial focuses on adult (18–30 year old) married, pregnant women (DILs) with a history of DV and their MILs. Research with pregnant women is typically considered to be in the high-risk category by institutional review boards, and if funded by the US government as in the case of this study, must meet the requirements laid out in the Common Rule [42]. Our formative research in Bengaluru suggested that promoting DV prevention as part of maternal and child health care was likely to be safer and more acceptable than one focused exclusively on DV. Indeed, Campbell has pointed out that the lack of DV screening and response in antenatal care settings is a huge missed opportunity for DV prevention [28]. This is especially the case in India where women face immediate, intense pressures to prove their fertility upon marriage; large proportions of women seek antenatal care (90% of urban married women had at least three antenatal examinations during their most recent pregnancy [20]); and women’s first experience of DV occurs within the initial few years of marriage [27].

**Table 2:** Eligibility criteria

**Daughters-in-law**	**MILs**
*Inclusion*	*Inclusion*
· Married	· MIL of a DIL who consents to participate
· 18–30 years	· Kannada or Tamil speaking
· Have ever experienced physical, psychological, or sexual violence perpetrated by husbands, MILs, or other family members	· Able and willing to provide consent
· Pregnant & in 1st or 2nd trimester^a^	
· Kannada or Tamil speaking	
· Able and willing to provide consent	
· Able and willing to refer MIL for study recruitment	
· DIL of a MIL who consents to participate	
*Exclusion*	*Exclusion*
· Cognitive impairment	· Cognitive impairment
· Planning to move or moving from the area prior to 6 months postpartum	· Planning to move or moving from the area before the DIL’s completion of 6 months postpartum

*Confirmed by urine pregnancy test and questions to assess stage of pregnancy (using the last menstrual period).

Reviewers of our research proposal strongly recommended that we screen DILs for a history of DV to ensure that women who are most likely to benefit from the intervention are recruited for the study. We have two reservations regarding this eligibility criterion. First, it is unclear whether women will be as likely to disclose DV during eligibility screening as they will in an interview setting after providing informed consent. Second, we feel that screening may be unnecessary given the high prevalence of DV among young married women in the study communities. On the other hand, focusing on women at highest risk of DV will ensure that we are best positioned to test the potential effectiveness of the intervention. Thus, we have decided to adhere to reviewers’ recommendation to screen potential participants for domestic conflict and violence.

### Recruitment and retention

We have designed a multistep recruitment, screening and informed consent process taking into account the need for family and community support, constraints in women’s decision-making autonomy and risks associated with discussions of DV. Based on feedback received from participants in our prior formative work, the study is framed in terms of a family health intervention that aims to promote the health of younger and older women and children. However, as recommended and approved by our Indian and US institutional review boards in order to ensure full disclosure, we will explain to DILs and MILs during informed consent that the study will address family conflict and violence and its health impacts. Street meetings and informal interactions will be conducted to introduce the study and the research team to women and their families, and to elicit broad support for the project. Community advisors comprising community elders and leaders identified during our previous study will be briefed about the project and given informational flyers. They will help convene community meetings and respond to questions that local residents may have.

In order to promote DILs’ decision-making, they will be contacted first and offered an opportunity to weigh the risks and benefits of participation before their MIL is contacted. DILs will be recruited through community outreach and at study centers. Staff will describe the study using a standard recruitment script and if women are interested in hearing more about the study and amenable to being screened, staff will administer a short screening questionnaire to assess eligibility (e.g., age, pregnancy status, status of MIL, language fluency, plans to move out of the community).

Interested and potentially eligible DILs will be invited to visit the center (if recruited in the community) for eligibility confirmation and informed consent. At the center, they will receive more detailed information regarding the study via verbal administration of the consent form, and then complete eligibility screening by taking a confirmatory pregnancy test and responding to questions related to their experience of family conflict and violence. Screening for family conflict and violence is planned to occur in the privacy of the study health center in order to facilitate disclosure. If eligible, DILs are invited to sign the informed consent form. DILs who consent will be able to enroll, contingent on their MIL’s consent for participation. They will be required to provide tracking/contact information to facilitate their MIL’s recruitment.

MILs will be referred to the health center by DILs who will be offered a copy of the study flyer to share with their MIL or, if DILs prefer, contacted by study staff, and similar screening and consent procedures will be implemented.

A Participant Tracking System will be used to store up-to-date information on participants’ address and fixed or mobile phone number, and track all contact attempts. Permission to use different tracking methods such as home visits, contacting friends/neighbors, and mobile phone calls will be obtained at enrollment and kept current. A key study aim is to identify retention challenges, which will be monitored quantitatively and qualitatively.

Study staff will be trained to be sensitive to the potential for breaches in confidentiality. During recruitment, they will closely monitor potential participants’ level of comfort, comprehension, and interest. If they detect discomfort, lack of interest, or lack of comprehension, they will either discontinue the process in an appropriate fashion or involve the field coordinator or study clinician. Similarly, care will be taken during participant follow-up. Staff will only speak to individuals that participants have granted prior permission to contact; avoid discussing any study specifics when trying to locate participants at home or work; and approach contacts in neutral terms; and use standardized scripts to respond to questions that arise in the community during this process. If participants choose to share sensitive information about themselves with study staff in a community setting, staff will encourage them to come to the health center to debrief in private and access services and support. Routine team debriefings will be conducted to review recruitment and follow-up related challenges.

### Safety monitoring

Monitoring and measuring safety of the *Dil Mil* intervention is challenging because of the high prevalence of family conflict and DV in this setting. Moreover, because the study plans to recruit young women with previous experience of family conflict and violence, safety monitoring will need to focus on identifying safety-related “events that reasonably might be linked to the intervention [12], p.116.”

Our team has received training to interact with participants in a sensitive and respectful manner and to facilitate disclosure of family conflict and violence. Staff will look for cues of emotional distress and provide emotional support to women who disclose DV. Both DILs and MILs will have the opportunity to meet one-on-one with an experienced counselor during regular clinic hours; appointments during non-clinic hours will be made if necessary. DILs will be encouraged to adhere to their antenatal visit schedule, and maternal and mental health assessments will be conducted to monitor their health during the pregnancy [43]. Antenatal visits will also provide an opportunity for reiteration of information regarding available support services.

On-site counseling and referral support will be made available, with information offered to participants verbally at each study visit, regardless of disclosure of victimization, and at an information counter that study staff have set up at the health centers. We have developed close relationships with support services such as child development agencies, shelters, and legal aid organizations in Bengaluru. Assistance such as escort to a referral care provider or an introductory letter or phone call will be provided to those who want to access these services and need support. Participants will be informed about how to access staff and support services at any point during the study. They will be instructed to contact the project director in the event of any threat to their safety or health due to study participation or other reasons. Referrals will be documented using a Referral Care Form and reviewed on a routine basis to ensure that appropriate care is being provided and to detect any potential study-related risks.

Building on previous intervention trials that have focused on vulnerable populations and included detailed risk appraisals (such as measures of depression and experience of physical harm), we established a safety monitoring system that centered around two types of safety-related events: safety alerts and adverse events. Safety alerts constitute “red flags.” They are events that may pose safety risks to participants, but may not involve participants directly, or if they do, are unlikely to be related to study participation or not considered to be severe. Examples of safety alerts include staff witnessing a family disagreement or conflict that is unrelated to the study, participant report of a minor altercation at home due to study participation, gossip in the community regarding study objectives, or enquiries about the study by family members. Miscarriages among DILs and illness episodes among MILs may be classified as safety alerts if they are determined to be unrelated to study participation. Safety alerts may warrant investigation by the study team to determine relatedness to study activities through discussion with participants and discreet gathering of information in the community (without jeopardizing participant confidentiality). Safety alerts may result in heightened monitoring of interactions with one or more participants, and will be reported to the IRB every six months.

Adverse events are defined as unexpected, unfavorable, and serious events such as serious injury, self-harm, hospitalization, or pregnancy loss that are temporally associated with and likely to be related to study participation. Adverse events will be reported to the IRB within 24 to 48 hours, and may result in substantive changes in the research protocol or other corrective actions in order to protect the safety, welfare, or rights of participants.

Definitions of safety alerts and adverse events were finalized through discussion and consensus among the study team and members of a two-person data and safety monitoring committee (DSMC) established for the study. Staff will be required to report all events without delay to the project director and principal investigators who will be responsible for classifying the event, developing appropriate responses, and reporting to the IRB and DSMC. All events will be recorded on an Incident Occurrence Reporting Form.

A range of resolutions may be pursued in the case of safety alerts and adverse events. These may include offering counseling or referrals, protocol modifications (e.g., change in participant tracking strategy), consultation with community advisors, and/or continued monitoring for recurrence of the event or occurrence among other participants. In our experience, responses to safety alerts need to be determined on a case-by-case basis, in consultation with the participant (when possible) and taking into account their best interests. Thus, our safety monitoring protocol describes guidelines for identifying events and allows for resolutions that are tailor-made to specific situations and cases.

The sensitivity of the issues we propose to address may place research staff in unsafe situations [15,44]. Our experienced staffs are highly trained to negotiate such situations and will have a safety plan to follow in the event of a challenge in the field. Staff safety strategies include working in pairs to recruit and follow-up participants, enlisting the support of community advisors or health center staff when appropriate, having access to a cell phone to contact other staff in case of an emergency, carrying identification badges, having timely and daily debriefs with the project director to discuss any situations of potential concern, and documenting any safety-related challenge/situation for further discussion and action.

### Randomization

Consenting dyads will be randomized as pairs on completion of the baseline assessment. Within each site, dyads will be randomized to ensure treatment balance after every 20 randomizations. The study statistician will employ a pseudo-randomizer software package to determine dyad treatment assignments and provide a series of sealed opaque randomization envelopes to study staff with one envelope per dyad. The participant IDs will be printed on the outside of the envelope, and the assigned study arm will be printed on a card contained in the envelope. A designated staff member at each center will open envelopes in sequential order of participant ID. Post randomization, the project director will ensure that the staff designated to facilitate intervention sessions do not conduct follow-up interviews or vice versa, and interviewers will not have access to information on treatment assignment.

### Intervention and control conditions

Dyads will be randomized to the control arm and receive standard care or to the intervention arm and receive standard care plus the *Dil Mil* intervention. Standard care for DILs includes primary care and access to DV-related support services [45,46]. For MILs, we will offer a comprehensive health examination including a gynecological exam and screening for diabetes and hypertension, along with appropriate information, prescriptions, and/or referrals. We will routinely offer DV-related resources, such as information, counseling, and referrals to all participants, DILs and MILs, regardless of study arm.

The intervention, which will be implemented during the second and third trimesters of the DILs’ pregnancy, consists of 2 half-day group sessions with DILs, 5 half-day group sessions with MILs, and one joint half-day session with DILs and MILs. *Dil Mil* is delivered at the health center to small groups consisting of 12 DILs or MILs (i.e., 6 groups of 12 individuals), except for the joint session in which DILs and their MILs participate together.

The curriculum is based on participatory learning and action principles and uses stories, role-play, and discussion to enhance participants’ knowledge, skills, and social support (see [36] for details). Facilitators will encourage participants to critically analyze their relationships and to develop and implement responses appropriate to their families and communities. The group education sessions with MILs will focus on the physical and psyschosocial dimensions of growing older and the impact of life experiences including experience of DV and familial relationships on their current health status. Based on these discussions, MILs will be encouraged to reflect on the health of daughters and DILs. Thus, by beginning and grounding discussions of DV and women’s health in personal experiences and by addressing MILs’ health concerns, we expect to reduce the risks of the intervention to participating DILs.

In addition, the joint MIL-DIL session, which is the most sensitive session, will focus on the promotion of infant health, including the creation of a violence-free home environment using case studies and role plays. Activities will be conducted to promote peer support and dialogue, such as co-counseling, which aims to build peer support and empathy through dyadic peer counseling interactions within the small education groups [47]. Facilitators will be trained to handle tense situations with sensitivity and equanimity (e.g., through the development and practice of using scripts to defuse or respond to specific scenarios). Since the joint session occurs after rapport has been independently built with DILs and MILs in separate sessions, there are sufficient reiterations of the goals of the intervention and the need to respect each other’s views.

To mitigate stress and worry related to childcare and household responsibilities during the time spent on study visits, we will offer childcare at the study centers. Women will be provided information and advance notice (through appointments) regarding study visits and therefore can make childcare and other household arrangements in advance. Finally, food (a meal and snacks) will be provided when women attend the sessions.

The risk of loss of confidentiality and privacy during the group education sessions will be minimized by ensuring that all participants are cognizant of the ground rules of participation in a group exercise, including the fact that they do not have to reveal their full name, and they should not reveal personal information about others but talk in general terms. At the end of the first session when ground rules are introduced, participants will be asked to take a pledge that they will not disclose personal information about other group members to people outside the group. At the beginning and end of each session, participants will be reminded that it is essential to respect the confidentiality of other members of the group and not to discuss other group members’ personal information outside of the meeting. However, participants will be told to keep in mind that the proceedings of the discussion may not be kept confidential, although all those present are encouraged to do so. During the joint sessions, standardized scripts will be used to sensitively reiterate the need for respect for different views that may arise during discussions.

### Sample size and power analysis

We conducted a power analysis based on the proposed sample size of 144 dyads. Our analysis of feasibility and safety (Aim 1) will use descriptive statistics and comparisons between arms without predetermined expectations regarding statistical power. In contrast, data related to Aims 2 and 3 will be analyzed using the intention to treat ( ITT) principle [48,49]. Power calculations, performed assuming a significance level of 0.05, 90% retention, and no contamination, indicated that we have sufficient statistical power to detect changes in intermediary outcomes , that is, indicators of DILs’ and MILs’ empowerment (e.g., proportion of DILs who report that wife beating is unjustified). If 45% of DILs in the control arm report that wife beating is unjustified [27], we will be able to detect a relative risk of 1.6 with 88% power. Due to resource and time constraints of a phase 2 trial, we did not power the trial to detect a significant effect on DV. Nevertheless, we estimated power calculations for DV among DILs during the first 6 months postpartum based on the literature. Given the proposed sample size, we will be able to detect a relative risk of 0.5 with power ranging from 23% to 95% when the proportion reporting DV in the control arm ranges from 20% to 50%.

### Quantitative assessments and analytic plan

Quantitative data on feasibility (including recruitment, retention, adherence, and contamination) and safety of the intervention will be gathered through study records pertaining to recruitment, adherence, retention and safety. Data on empowerment of DILs and Mils (knowledge about safety and the links between DV and health, gender-equitable attitudes, decision-making skills, communication and social support, and resistance to DV) and incidence of DV and related health outcomes (e.g., quality of life, anxiety and depression, and maternal and infant health) will be gathered by research interviewers not involved in intervention implementation through face-to-face interviews. Interviews with DILs and MILs will be conducted individually at enrollment and 3 months and 6 months postpartum. Measures were chosen based on our theoretical approach and drawn from instruments used in our previous research, the Indian National Family Health Survey and studies of GBV and health in India and elsewhere (Table 3). Questions were selected keeping in mind the need to minimize study participation-related risks. For example, questions regarding MILs’ perpetration of violence will only be posed to DILs, and MILs’ experience of DV will also be posed in order to monitor safety of the intervention.

**Table 3:** Measures

**Measure**	**DIL**	**MIL**
**Feasibility and Safety**		
Recruitment, adherence, retention (proportion of eligible individuals who enroll, sociodemographic comparison of enrollees and non-enrollees, adherence to intervention and data collection visits, attrition)	X	X
Safety (e.g., number and nature of safety-related events)	X	X
Contamination (knowledge of neighbors, friends, or relatives participating in the intervention arm)	X^a^	X^a^
**Individual-level Indicators of Empowerment**		
Demographic indicators of access to resources (e.g., age, marital duration, parity, education, employment, socioeconomic status, household structure and husband’s age, education and income [27])	X	X
Knowledge and perceptions of DV & family Health (e.g., knowledge and perceptions of reproductive and infant health [27], links between DV and health)	X	X
Gender-equitable attitudes (modified version of the Gender Equitable Male [GEM] scale [50])	X	X
Decision-making (involvement in household decisions [27], DILs’ involvement in food-related decision-making, household food insecurity access scale [51])	X	X
Freedom of movement (indicators of mobility [52])	X	X
**Interpersonal- and Family-level Indicators of Empowerment**		
Social support (DILs’ perceptions of social support and MILs’ provision of emotional and practical support using an adapted Multidimensional Scale of Perceived Social Support [53])	X	X
Communication (indicators of communication between MILs and DILs and with sons/husbands regarding household matters)	X	X
Resistance to violence (DILs’ overt and covert resistance to DV and MILs’ opposition to DV by their son)	X	X
**Outcomes**		
Domestic violence (measures of physical, sexual, and psychological acts of violence perpetrated by husbands, MILs, or other family members using an adapted Conflict Tactics Scale [54])	X	X
Perceived quality of life (WHOQOL-BREF [55])	X	X
Anxiety and depression (Kessler Psychological Distress Scale – K10)	X	X
Postnatal Depression (Edinburgh Postnatal Depression Scale [56])	X	
Maternal and Infant Health (maternal height, weight, weight gain during pregnancy, and BMI; infant weight, length, and head circumference)	X	

^a^Assessed only among control arm MILs and DILs.

Descriptive analyses will be conducted on data related to Aim 1 while data for Aims 2 and 3 will be analyzed using the intention to treat approach. Appropriate statistical techniques will be used to compare study arms on outcomes of interest (DILs’ and MILs’ empowerment, DV incidence, quality of life, anxiety and depression and maternal and infant health status) and, tailored by whether the outcome is binary, categorical, or continuous. Analytic models of intervention effect will include adjustments for individual-level demographic and background variables, such as age, education, and socioeconomic status, measured at baseline prior to randomization to examine for improved precision and to adjust for any (random) covariate imbalance across groups [57]. Longitudinal measurements of outcomes will be exploited using statistical techniques for matched or longitudinal analysis (matched pair analysis, before-after comparisons, generalized estimating equations for longitudinal models) to assess whether additional precision in efficacy estimates is possible. Finally, in the intervention group, we will explore the association between number of sessions attended by MILs and DILs and DV incidence to examine possible dose–response.

Prior to estimating program effects, simple statistics (e.g., means, variances, frequencies, correlations) will be examined to characterize DILs and MILs on various measures (including the experience of different types of DV by perpetrator) and to summarize indicators of feasibility and safety. The distributional properties and reliability of continuously scaled variables will be examined. We will verify that intervention and control groups do not differ on important baseline characteristics that would complicate the evaluation. Bivariable relationships will be examined using chi-square tests for categorical variables and two sample t-tests or Wilcoxon Rank Sum tests, as appropriate, for continuous variables. Highly collinear (r>0.5) variables will be identified so as not to be included together in subsequent analyses.

### Qualitative assessments and analytic plan

We will gather in-depth information on study implementation-related issues that complement the quantitative data through three sets of 10 in-depth interviews (n=30) after the 6-month postpartum evaluation [58]. They include the following: (1) To better understand participants’ experience with and feedback on the intervention and its effectiveness, we will select, from the intervention arm, 5 DILs and 5 MILs who participated in all intervention sessions for an in-depth interview. These data will contribute to the refinement of the intervention curriculum to maximize its potential impact on DV. (2) To assess logistical, DV-related, or other barriers to intervention adherence and retention for improving a future phase 3 trial, we will interview 10 intervention arm participants who did not attend all scheduled sessions (that is, MILs who attended fewer than 5 sessions and DILs who attended only 1 or 2 out of 3 sessions, assuming they are not lost to follow-up). (3) To examine the presence of contamination, we will interview 10 DILs and MILs in representative proportions drawn from those control arm participants who reported knowing neighbors, friends, or relatives participating in the intervention arm at the 6-month postpartum interview. These interviews will focus on assessing extent of knowledge regarding the content of the intervention and ways in which this information may have influenced participants’ perspectives on gender equity and family relationships and perpetration and experience of DV.

In-depth interviews will be audio-taped, transcribed verbatim, translated into English, and imported into ATLAS.ti®. Analysis will be carried out using the following steps: (1) data immersion through repeated readings of transcripts and associated field notes until content familiarity is high; (2) summarization of key themes that emerge in the narratives in the form of theoretical and analytical memos for each major category; (3) development of codes and a codebook with definitions through team discussion of key themes and based on memos, using a combination of inductive and deductive approaches; the coding structure will be hierarchically ordered so that additional dimensions of meaning can be examined separately and so that new codes can be added as data collection and analysis progress; (4) coding by two senior staff, using steps described by Carey et al. [59], to ensure intercoder agreement; (5) generation of meaning from coded data by examining variation within each theme/code and differences between individuals/subgroups; comparison and categorization will be facilitated by the summarization of data through diagrams and tables; and (6) interpretations and key lessons regarding intervention quality, feasibility and adherence, and contamination extracted through the above process and discussion within the investigative team [60-62].

## Discussion

This study will evaluate the feasibility, safety, and potential effectiveness of an innovative women’s empowerment intervention to mitigate DV and related adverse health outcomes. The intervention has been developed on the basis of our past research in India and successful experiences in DV prevention in other parts of the world. The planned phase 2 trial will enable us to evaluate the feasibility, safety, and potential effectiveness of the intervention and is a necessary prerequisite for a phase 3 trial, providing evidence critical to its design, including preliminary data on effect sizes and the potential for contamination.

In our phase 1 pilot study, we established the acceptability of the *Dil Mil* intervention and demonstrated that it is likely safe. However, due to the limited sample size and participant follow-up, safety concerns remain. Empowering young women can result in backlash from their husband and his family. Although the risk for backlash is minimized by the inclusion and empowerment of MILs, a careful study of safety is warranted. The *Dil Mil* trial has been designed keeping in mind research-related risks and incorporating an array of strategies to reduce these risks as well as to monitor and measure safety. Thus, this study offers approaches that may help guide safety planning and monitoring in other domestic violence intervention trials in similar settings. Moreover, given the staggeringly high prevalence of DV among young women in India (and indeed globally) and the dearth of data on effective interventions, this study is poised to make an important contribution to the evidence-base for DV prevention.

Enrolling pregnant women is a compelling approach because of the growing utilization of primary health care and especially antenatal services in India, the fact that women in India tend to marry young and bear children early in their relationship, and the acceptability of DV prevention as part of maternal and child health promotion. Although testing our intervention in this population will limit the generalizability of our results, we believe that the strengths of this approach outweigh the attendant limitation.

As is typical of phase 2 trials, we will have limited statistical power to evaluate the effect of the intervention on DV, but substantially greater statistical power to examine the effect on intermediary outcomes (DILs’ and MILs’ empowerment). These results will provide important insights into whether and how the intervention might have an effect.

Our study does have several limitations. Evaluations of empowerment-based DV interventions on which *Dil Mil* is based indicate that the effect of the intervention at least on intermediary outcomes will be manifest by 6 months postpartum [38,40]. We recognize that a longer follow-up might be preferable for a future, more resource-intensive phase 3 trial. Further, DIL-MIL dyads enrolled and retained in this study may represent those who are at relatively lower risk of DV. We will elicit qualitative feedback from participants on recruitment strategies to improve our ability to reach the most vulnerable young women in the future. In our prior longitudinal study, women with a history of DV were no more likely to be lost to follow-up than other women (ref), indicating that we will be able to retain this vulnerable group. Finally, because we are randomizing dyads in select geographic areas, contamination between study arms may occur, increasing chances of dilution of the treatment effect (a type ll error). As part of study Aim 1, we will assess the presence of contamination quantitatively and qualitatively and consider the need to use an alternative design such as cluster randomization for a future phase 3 trial.

In conclusion, this study will provide quantitative and qualitative evidence to determine whether a phase 3 effectiveness trial of the *Dil Mil* intervention is merited. Criteria in favor of a phase 3 trial include quantitative and qualitative data indicating that the intervention is safe and feasible; data on intermediary outcomes support the effect of the intervention on empowerment of DILs and MILs; and trends in GBV incidence and related health outcomes are in the direction that supports the effect of the intervention.

## Competing interests

The authors declare that they have no competing interests.

## Authors’ contributions

All authors have made an intellectual contribution to this study. SKrishnan conceived of the study, oversaw its design and implementation, and drafted the manuscript. SKrishnamachari participated in study design and coordination and helped to draft the manuscript. PC and NP contributed to the design of the intervention and the trial and the identification of research questions. KS contributed to the design of the intervention, managed study implementation and helped to draft the manuscript. All authors have read and approved the final manuscript.

## Pre-publication history

The pre-publication history for this paper can be accessed here:

http://www.biomedcentral.com/1471-2458/12/943/prepub

## References

[B1] Rocca C Rathod S Falle T Pande R Krishnan S Challenging assumptions about women’s empowerment: social and economic resources and domestic violence among young married women in urban South India Int J Epidemiol 2009 38 2 577 585 18952621 10.1093/ije/dyn226 PMC2734072

[B2] International Institute for Population Sciences (IIPS) and Macro International National Family Health Survey (NFHS 3), 2005-06: Fact Sheet Karnataka 2007 Mumbai: IIPS 1 3

[B3] Ackerson LK Kawachi I Barbeau EM Subramanian SV Effects of Individual and Proximate Educational Context on Intimate Partner Violence: A Population-Based Study of Women in India Am J Public Health 2008 98 3 507 514 10.2105/AJPH.2007.113738 18235066 PMC2253590

[B4] Ackerson LK Subramanian SV Domestic Violence and Chronic Malnutrition among Women and Children in India Am J Epidemiol 2008 167 10 1188 1196 10.1093/aje/kwn049 18367471 PMC2789268

[B5] Chandran M Tharyan P Muliyil J Abraham S Post-partum depression in a cohort of women from a rural area of Tamil Nadu, India - Incidence and risk factors Br J Psychiatry 2002 181 499 504 10.1192/bjp.181.6.499 12456520

[B6] Jejeebhoy SJ Associations between wife-beating and fetal and infant death: Impressions from a survey in rural India Stud Fam Plan 1998 29 3 300 308 10.2307/172276 9789323

[B7] Jejeebhoy SJ Cook RJ State accountability for wife-beating: The Indian challenge Lancet 1997 349 S10 S12 10.1016/s0140-6736(97)90004-0 9057772

[B8] Jeyaseelan LK Neelakantan S Peedicayil N Pillai R Duvvury N Physical spousal violence against women in India: some risk factors J Biosoc Sci 2007 39 657 670 10.1017/S0021932007001836 17349066

[B9] Koenig MA Stephenson R Ahmed S Jejeebhoy SJ Campbell J Individual and contextual determinants of domestic violence in north India Am J Public Health 2006 96 1 132 138 10.2105/AJPH.2004.050872 16317213 PMC1470450

[B10] Martin SL Mackie L Kupper LL Buescher P Moracco KE Physical abuse of women before, during, and after pregnancy JAMA 2001 285 12 1581 1584 10.1001/jama.285.12.1581 11268265

[B11] Silverman J Decker M Kapur N Gupta J Raj A Violence against wives, sexual risk and sexually transmitted infection among Bangladeshi men Sex Transm Infect 2007 83 211 215 17301104 10.1136/sti.2006.023366 PMC2659096

[B12] Czaja SJ Schulz R Belle SH Burgio LD Armstrong N Gitlin LN Coon DW Martindale-Adams J Klinger J Stahl SM Data and safety monitoring in social behavioral intervention trials: the REACH II experience Clin Trials 2006 3 2 107 118 10.1191/1740774506cn136oa 16773953 PMC1484572

[B13] NIMH Multisite Prevention Trial Definition of adverse reactions in clinical trials of a behavioral intervention AIDS 1997 11 Suppl 2 S55 57 9475712

[B14] World Health Organization Putting women's safety first: Ethical and safety recommendations for research on domestic violence against women 2001 Geneva: WHO 1 32

[B15] Ellsberg M Heise L Bearing witness: ethics in domestic violence research Lancet 2002 359 9317 1599 1604 10.1016/S0140-6736(02)08521-5 12047984

[B16] Andersson N Cockcroft A Ansari N Omer K Chaudhry UU Khan A Pearson L Collecting reliable information about violence against women safely in household interviews: experience from a large-scale national survey in South Asia Violence Against Women 2009 15 4 482 496 10.1177/1077801208331063 19211909

[B17] Jewkes R Watts C Abrahams N Penn-Kekana L Garcia-Moreno C Ethical and methodological issues in conducting research on gender-based violence in Southern Africa Reprod Health Matters 2000 8 15 93 103 10.1016/S0968-8080(00)90010-7 11424273

[B18] Sikweyiya Y Jewkes R Perceptions and experiences of research participants on gender-based violence community based survey: implications for ethical guidelines PLoS One 2012 7 4 e35495 10.1371/journal.pone.0035495 22558160 PMC3338721

[B19] Fontes LA Ethics in violence against women research: The sensitive, the dangerous, and the overlooked Ethics & Behavior 2004 14 2 141 174 10.1207/s15327019eb1402_4 15835038

[B20] District Level Household and Facility Survey 2007–08: Fact Sheets - India 2007 [http://www.rchiips.org/pdf/rch3/state/India.pdf]

[B21] Child Marriage Fact Sheet [http://www.unicef.org/india/Child_Marriage_Fact_Sheet_Nov2011_final.pdf]

[B22] Barua A Kurz K Reproductive health-seeking by married adolescent girls in Maharashtra, India Reprod Health Matters 2001 9 17 53 62 10.1016/S0968-8080(01)90008-4 11468846

[B23] Dyson T Moore M On Kinship Structure, Female Autonomy, and Demographic Behavior in India Popul Dev Rev 1983 9 1 35 60 10.2307/1972894

[B24] Goyal RS Dimensions of adolescent motherhood in India Soc Biol 1994 41 1–2 130 134 7973838 10.1080/19485565.1994.9988865

[B25] Jejeebhoy S Sebastian M Actions that protect: promoting sexual and reproductive health and choice among young people in India South and East Asia Regional Working Papers No 18 2003 New Delhi, India: Population Council http://www.popcouncil.org/pdfs/wp/seasia/seawp18.pdf

[B26] Krishnan S Rocca C Hubbard A Subbiah K Edmeades J Padian NS Do Changes in Spousal Employment Status Lead to Domestic Violence? Insights from a Prospective Study in Bangalore, India Soc Sci Med 2009 70 1 136 143 19828220 10.1016/j.socscimed.2009.09.026 PMC2791993

[B27] International Institute for Population Sciences (IIPS) and Macro International National Family Health Survey (NFHS 3), 2005-06: India, volume 1 2007 Mumbai: IIPS

[B28] Campbell JC Abuse during pregnancy: a quintessential threat to maternal and child health - so when do we start to act? Can Med Assoc J 2001 164 11 1578 1579 11402797 PMC81113

[B29] Coker AL Does physical intimate partner violence affect sexual health? A systematic review Trauma Violence Abuse 2007 8 2 149 177 10.1177/1524838007301162 17545572

[B30] Koenig LJ Whitaker DJ Royce RA Wilson TE Ethier K Fernandez MI Physical and sexual violence during pregnancy and after delivery: A prospective multistate study of women with or at risk for HIV infection Am J Public Health 2006 96 6 1052 1059 10.2105/AJPH.2005.067744 16670222 PMC1470613

[B31] Muthal-Rathore A Tripathi R Arora R Domestic violence against pregnant women interviewed at a hospital in New Delhi Int J Gynaecol Obstet 2002 76 1 83 85 10.1016/S0020-7292(01)00533-1 11818101

[B32] Purwar MB Jeyaseelan L Varhadpande U Motghare V Pimplakute S Survey of physical abuse during pregnancy GMCH, Nagpur, India J Obstet Gynaecol Res 1999 25 3 165 171 10.1111/j.1447-0756.1999.tb01142.x 10467788

[B33] Varma D Chandra PS Thomas T Carey MP Intimate partner violence and sexual coercion among pregnant women in India: Relationship with depression and post-traumatic stress disorder J Affect Disord 2007 102 1–3 227 235 17109969 10.1016/j.jad.2006.09.026 PMC1978173

[B34] Krishnan S Iyengar S Pande RP Subbiah K Roca E Anuradha R Measham D Marriage and Motherhood: Influences on Women’s Power in Sexual Relationships Population Association of America, 2005 Annual Meeting. Philadelphia 2005

[B35] Staying Alive: 5th Monitoring & Evaluation 2012 on the Protection of Women from Domestic Violence Act 2005 [http://www.lawyerscollective.org/files/Staying%20Alive%205th%20M&E.pdf]

[B36] Krishnan S Subbiah K Khanum S Chandra PS Padian NS An intergenerational women's empowerment intervention to mitigate domestic violence: results of a pilot study in Bengaluru, India Violence Against Women 2012 18 3 346 370 10.1177/1077801212442628 22531083

[B37] Parker B McFarlane J Socken K Silva C Reel S Testing an intervention to prevent further abuse to pregnant women Res Nurs Heal 1999 22 59 66 10.1002/(SICI)1098-240X(199902)22:1<59::AID-NUR7>3.0.CO;2-B 9928964

[B38] Pronyk PM Hargreaves JR Kim JC Morison LA Phetla G Watts C Busza J Porter JDH Effect of a structural intervention for the prevention of intimate-partner violence and HIV in rural South Africa: a cluster randomised trial Lancet 2006 368 9551 1973 1983 10.1016/S0140-6736(06)69744-4 17141704

[B39] Tiwari A Leung WC Leung TW Humphreys J Parker B Ho PC A randomised controlled trial of empowerment training for Chinese abused pregnant women in Hong Kong BJOG 2005 112 9 1249 1256 10.1111/j.1471-0528.2005.00709.x 16101604

[B40] Bangalore (Bengaluru) Cityhttp://www.census2011.co.in/census/city/448-bangalore.html

[B41] Civic Health Services Guide http://www.bmponline.org/forms-pubs/PUBs/BMP-HE-E-AUG03-P-106.pdf

[B42] Code of Federal Regulations: Title 45 Public Welfare - Part 46 Protection of Human Subjectshttp://www.hhs.gov/ohrp/policy/ohrpregulations.pdf 11686173

[B43] Satyanarayana VA Chandra PS Should mental health assessments be integral to domestic violence research? Indian J Med Ethics 2009 VI 1 15 18 19241949 10.20529/IJME.2009.004 PMC2898270

[B44] Logan TK Walker R Shannon L Cole J Combining ethical considerations with recruitment and follow-up strategies for partner violence victimization research Violence Against Women 2008 14 11 1226 1251 10.1177/1077801208323976 18809849 PMC11959690

[B45] Government of India Indian Public Health Standards for Primary Health Centres New Delhi: Directorate General of Health Services Ministry of Health & Family Welfare 2006 http://mohfw.nic.in/NRHM/Documents/IPHS_for_PHC.pdf

[B46] World Health Organization Antenatal Care Randomized Trial: Manual for the Implementation of the New Model 2001 Geneva: World Health Organization http://whqlibdoc.who.int/hq/2001/WHO_RHR_01.30.pdf

[B47] Kauffman K New C Co-counselling: the theory and practice of re-evaluation counselling 2004 New York, NY: Brunner-Routledge

[B48] Begg C Ruminations on the Intent-to-Treat Principle Control Clin Trials 2000 21 3 241 243 10.1016/S0197-2456(00)00050-7 10822121

[B49] Lachin J Statistical Considerations in the Intent-to-Treat Principle Control Clin Trials 2000 21 2 167 189 10822117 10.1016/s0197-2456(00)00046-5

[B50] Verma RK Pulerwitz J Mahendra V Khandekar S Barker G Fulpagare P Singh SK Challenging and changing gender attitudes among young men in Mumbai, India Reprod Health Matters 2006 14 28 135 43 10.1016/S0968-8080(06)28261-2 17101432

[B51] Swindale A Bilinsky P Development of a universally applicable household food insecurity measurement tool: process, current status, and outstanding issues J Nutr 2006 136 Suppl 1449S 52S 16614442 10.1093/jn/136.5.1449S

[B52] Jejeebhoy S Women's autonomy and reproductive behavior in India: linkages and influence of sociocultural context Comparative Perspectives on Fertility Transition in South Asia: Volume I 1996 Liege, Belgium: International Union for the Scientific Study of Population 20 26

[B53] Zimet G Dahlem N Zimet S Farley G The Multidimensional Scale of Perceived Social Support J Pers Assess 1988 52 1 30 41 10.1207/s15327752jpa5201_2

[B54] Garcia-Moreno C Jansen HAFM Ellsberg M Heise L Watts C WHO Multi-country Study on Women’s Health and Domestic Violence against Women. Initial results on prevalence, health outcomes and women’s responses 2005 Geneva: World Health Organization

[B55] World Health Organization WHOQoL Study Protocol 1993 Geneva: WHO

[B56] Benjamin D Chandramohan A Annie I Prasad J Jacob K Validation of the Tamil version of Edinburgh post-partum depression scale J Obstet Gynaecol India 2005 55 3 241 3

[B57] Rubin DB van der Laan M Covariate Adjustment for the Intention-to-Treat Parameter with Empirical Efficiency Maximization Working Paper Series. Working Paper 229 2008 UC Berkeley: Division of Biostatistics [http://biostats.bepress.com/ucbbiostat/paper229/] PMC2669310 19381345

[B58] Greene JC Caracelli VJ Graham WF Toward a conceptual framework for mixed-method evaluation designs Educ Eval Pol 1989 11 3 255 274

[B59] Carey JW Morgan M Oxtoby MJ Intercoder agreement in analysis of responses to open-ended interview questions: Examples from tuberculosis research Cultural Anthropology Methods 1996 8 3 1 5

[B60] Miles M Huberman A Qualitative Data Analysis: An Expanded Sourcebook 1994 Thousand Oaks, CA: Sage Publications

[B61] Strauss A Corbin JM Grounded Theory Methodology: An Overview Handbook of Qualitative Research 1994 Newbury Park, CA: Sage Publications

[B62] Weitzman E Miles M Computer Programs for Qualitative Data Analysis: A Software Sourcebook 1995 Thousand Oaks, CA: Sage Publications

